# Cytotrophoblast, Not Syncytiotrophoblast, Dominates Glycolysis and Oxidative Phosphorylation in Human Term Placenta

**DOI:** 10.1038/srep42941

**Published:** 2017-02-23

**Authors:** Kevin S. Kolahi, Amy M. Valent, Kent L. Thornburg

**Affiliations:** 1 Department of Biomedical Engineering, Oregon Health and Science University, Portland, OR 97239 USA; 2 Center for Developmental Health, Knight Cardiovascular Institute Oregon Health and Science University, Portland, OR 97239 USA; 3 Department of Obstetrics and Gynecology, Oregon Health and Science University, Portland, OR 97239 USA

## Abstract

The syncytiotrophoblast (SCT) at the maternal-fetal interface has been presumed to be the primary driver of placental metabolism, and the underlying progenitor cytotrophoblast cells (CTB) an insignificant contributor to placental metabolic activity. However, we now show that the metabolic rate of CTB is much greater than the SCT. The oxygen consumption and extracellular acidification rate, a measure of glycolysis, are both greater in CTB than in SCT *in vitro* (CTB: 96 ± 16 vs SCT: 46 ± 14 pmol O_2_ × min^−1^ × 100 ng DNA^−1^, p < 0.001) and (CTB: 43 ± 6.7 vs SCT 1.4 ± 1.0 ∆mpH × min^−1^ × 100 ng DNA^−1^, p < 0.0001). Mitochondrial activity, as determined by using the mitochondrial activity-dependent dye Mitotracker CM-H_2_TMRosa, is higher in CTB than in SCT in culture and living explants. These data cast doubt on the previous supposition that the metabolic rate of the placenta is dominated by the SCT contribution. Moreover, differentiation into SCT leads to metabolic suppression. The normal suppression of metabolic activity during CTB differentiation to SCT is prevented with a p38 MAPK signaling inhibitor and epidermal growth factor co-treatment. We conclude that the undifferentiated CTB, in contrast to the SCT, is highly metabolically active, has a high level of fuel flexibility, and contributes substantially to global metabolism in the late gestation human placenta.

The primary function of the human placenta is to ensure that the fetus it serves continuously receives maternally-derived nutrients that are necessary for optimal growth. The placenta is a powerful organ that produces hormones, alters nutrients biochemically as needed, and transports them to the developing fetus. Each of these processes exacts a metabolic cost. Thus, the placenta has an extraordinarily high metabolic rate, consuming approximately 40% of the oxygen used by the entire conceptus while accounting for less than 20% of its mass^1^,^2^,^3^,^4^. The metabolic status of the placenta is evidently important because several maternal disorders including pre-eclampsia and gestational diabetes mellitus, are associated with metabolic abnormalities^5^,^6^,^7^.

The materno-placental barrier is composed of a monolayer of terminally differentiated trophoblast cells, the syncytialized trophoblast (SCT), the underlying layer of progenitor cells, the cytotrophoblast (CTB) and the endothelium of the fetal vasculature. Throughout gestation, the SCT is continually repaired by CTB cells^8^. The SCT has been presumed to account for the largest proportion of placental metabolic activity because it is in direct contact with maternal blood, synthesizes and secretes large quantities of protein and steroid hormones, and is the primary transport organ for all nutrients acquired by the fetus. Furthermore, it has been assumed that because the CTB layer gradually disappears with gestational age, it plays an increasingly diminished biological role as term approaches^9^.

Freshly isolated CTB and SCT can be studied *in vitro*. Isolated CTB are known to differentiate and fuse over days of time in culture whereupon they form a multinucleated syncytium, presumably mimicking differentiation *in vivo*^10^,^11^. CTB that has been freshly isolated from normal human placenta^10^ will differentiate and fuse to form multinucleated SCT over 72 h in culture. Using this culture system, we recently discovered that CTB cells rapidly esterify a long chain BODIPY-labeled free-fatty acid. This finding cast uncertainty on the prevailing view that most nutrient processing is restricted to the SCT^12^. Not only is the free fatty acid esterification process performed within the CTB in cultured cells and living explants, but CTB also rapidly generate lipid droplets containing triglycerides and other lipid species including phospholipids. We also found that a host of genes responsible for the regulation of lipid uptake and metabolism are highly expressed in CTB but are suppressed as CTB differentiates into SCT^12^. Esterification of free-fatty acids in CTB poses a high ATP cost^13^, suggesting that the CTB may have higher metabolic needs than previously thought.

The metabolic contributions made by each of the many cell types in the placenta have not been well studied^14^. Thus, for this study, we tested the hypothesis that CTB is more metabolically active than SCT and more capable of generating ATP. To thoroughly examine the metabolic properties of the two trophoblast cell types, we compared their capacity for oxidative phosphorylation and glycolysis with different fuel substrates, and measured their intracellular ATP levels. We also tested the hypothesis that activity-sensitive mitochondrial dyes could distinguish the two trophoblast cell types using optical analyses.

## Results

**Figure 1:**
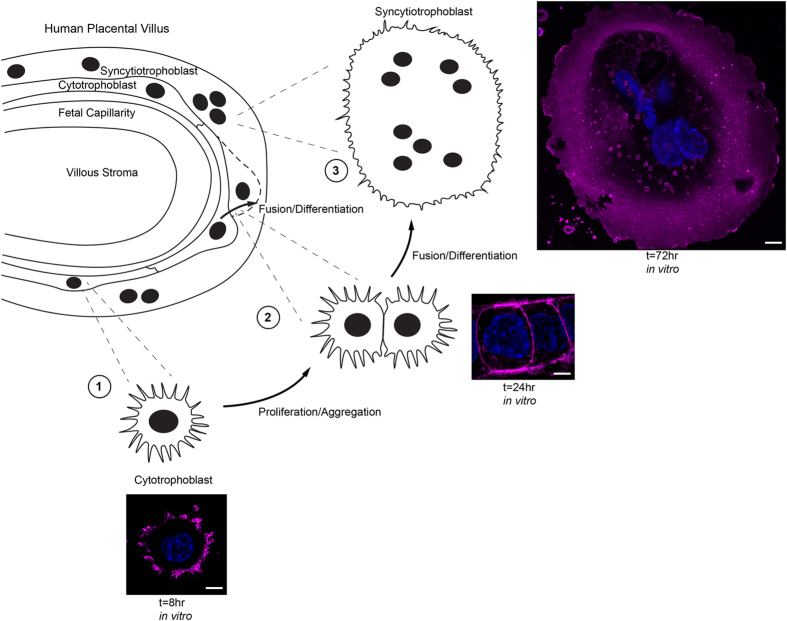
Isolated cytotrophoblast cells fuse and differentiate to form syncytiotrophoblast *in vitro*. The cellular cytotrophoblast (CTB), a progenitor epithelium which regenerates the syncytiotrophoblast (SCT) continuously, can be isolated and cultured *in vitro*. The CTB then recapitulates developmental differentiation as it becomes SCT. Wheat germ agglutinin (magenta) was used to label plasma membrane; nuclei are labeled with Hoechst dye (blue). (1) At initial plating, CTB are round and contain numerous filopodial extensions. (2) By 24 h of culture CTB begin to aggregate and form intercellular contacts in preparation for fusion. (3) By 72 h in culture, virtually all isolated CTB have fused to form SCT. The regeneration of SCT from CTB in cultured placental explants follows a similar time course^11^. Scale Bar: 5 μm.

We studied isolated CTB cells cultured for 8 and 72 h, representing undifferentiated CTB and differentiated SCT, respectively (Fig. 1).

**Figure 2:**
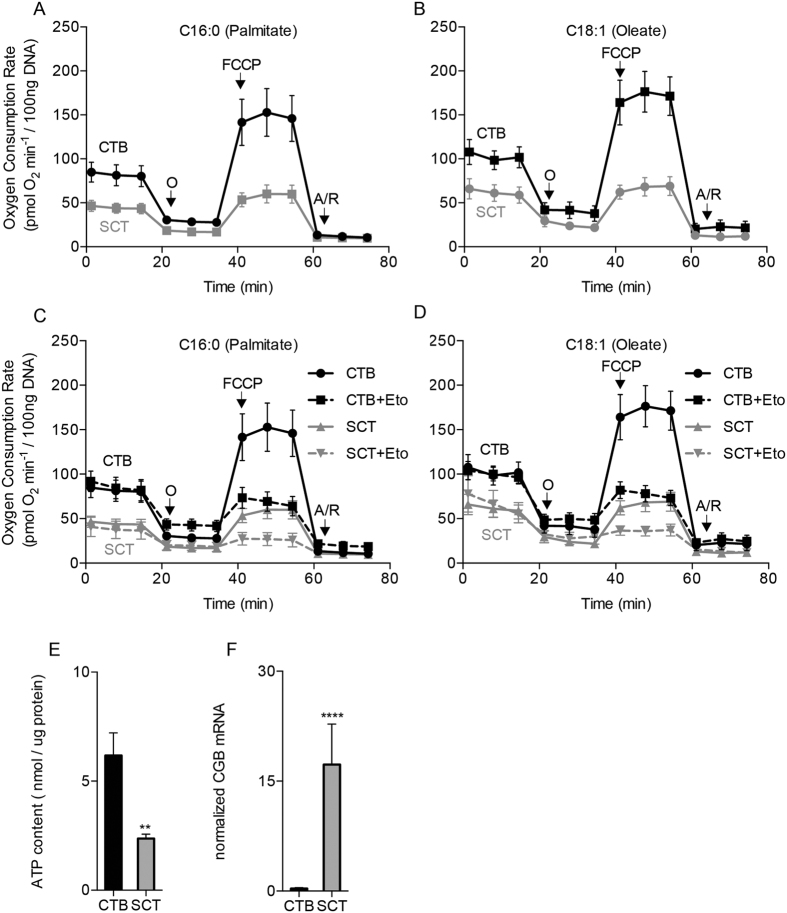
Human trophoblast respiration rates. Human cytotrophoblast cells (CTB) were isolated from term placentas and studied *in vitro* before and after differentiation into syncytiotrophoblast (SCT). (**A**,**B**) Respiration was measured in Krebs-Henseleit buffer containing 250 μM of either saturated long-chain fatty acid palmitate (C16) or the monounsaturated oleate (C18) using the Seahorse XF Analyzer. Oxygen consumption rate (OCR) was greater in CTB than in SCT at baseline (0 to15 min), at maximal oxidative phosphorylation with FCCP (40–60 min) and at all other points during a standard mitochondrial stress protocol. (**C**,**D**) Etomoxir, an inhibitor of carnitine palmitoyltransferase-1 (CPT1a), reduced maximal OCR, measured after FCCP injection, in both CTB and SCT for both fatty acids but not at baseline. (**E**) Resting ATP levels were also much higher in CTB than in SCT. (**F**) We measured the SCT marker, chorionic gonadotropin beta (CGB) and showed that it was highly expressed at the 72 hr time point (SCT) but not at 8 hr (CTB). Oligomycin (O), Antimycin/Rotenone (A/R). Data are Mean ± SEM, n = 7 placentas.

Isolated CTB had a greater oxygen consumption rate (OCR) at baseline and at maximal capacity than did SCT in the presence of long-chain fatty acid; Palmitate (C16) [baseline: 85 ± 11 vs 47 ± 6 pmol O_2_ × min^−1^ × 100 ng DNA^−1^, p < 0.01; maximal capacity: 153 ± 27 vs 60 ± 10 pmol O_2_ × min^−1^ × 100 ng DNA^−1^, p < 0.001 (Fig. 2A)] or Oleate (C18:1) [baseline: 108 ± 14 vs 66 ± 11 pmol O_2_ × min^−1^ × 100 ng DNA^−1^, p < 0.001; maximal capacity: 176 ± 23 vs 68 ± 11 pmol O_2_ × min^−1^ × 100 ng DNA^−1^, p < 0.0001 (Fig. 2B)].

Treatment with Etomoxir, an inhibitor of Carnitine Palmitoyl-transferase 1a, reduced maximal OCR in CTB and after it differentiates to SCT in the presence of both fatty acids [Palmitate (C16) 69 ± 11 pmol O_2_ × min^−1^ × 100 ng DNA^−1^, p < 0.0001; Oleate (C18:1) 78 ± 9.2 pmol O_2_ × min^−1^ × 100 ng DNA^−1^, p < 0.0001 (Fig. 2C,D)] and SCT [Palmitate (C16) 27 ± 7.3 pmol O_2_ × min^−1^ × 100 ng DNA^−1^, <0.0001; Oleate (C18:1): 36 ± 5.4 pmol O_2_ × min^−1^ × 100 ng DNA^−1^, p < 0.0001 (Fig. 2C,D)]. Intracellular ATP levels were higher in CTB compared to SCT [6.2 ± 2.9 vs 2.4 ± 0.55 nmol × ug protein^−1^; p < 0.01 (Fig. 2E)]. It has been previously established that SCT is the placental cell for steroid and chorionic gonadotropin production. Because chorionic gonadotropin beta (CGB) is produced by SCT but not by CTB, it is a reliable marker for trophoblast differentiation [5933 ± 150%; p < 0.0001 (Fig. 2F)].

Human trophoblasts have specialized free-fatty acid uptake systems to serve as the selective conduit for maternal long- and very-long chain polyunsaturated fatty acids. Since our assay media contained primarily fatty acids as a fuel source, we wondered whether the greater OCR in CTB could be a byproduct of their greater free-fatty acid uptake capacity whereas SCT may selectively metabolize other fuels.

**Figure 3:**
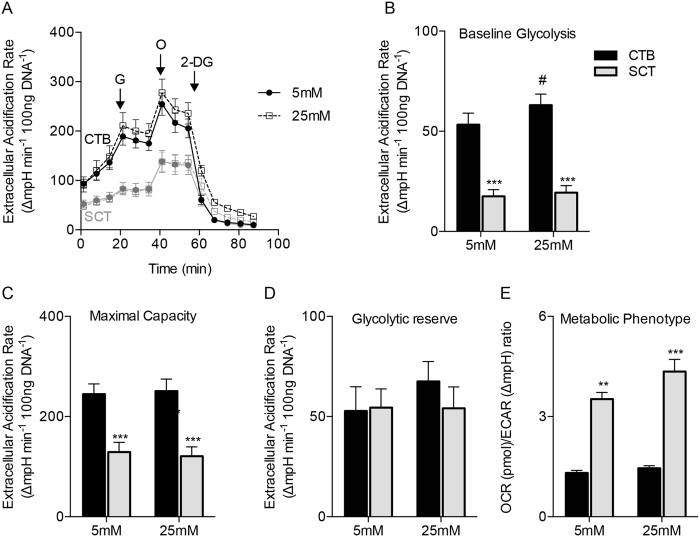
Cytotrophoblast are highly glycolytic. The glycolytic metabolism of primary human trophoblast cells was measured *in vitro* using the Seahorse XF Analyzer. (**A**) CTB and SCT extracellular acidification rates (ECAR), a metric of glycolytic lactate production, was measured during a glycolytic stress experiment with Seahorse assay medium that was supplemented with pyruvate and glutamine only. At point G, 5 mM or 25 mM glucose was injected, 1 μM oligomycin at point O, and 100 mM 2-Deoxyglucose at point 2-DG. Oligomycin inhibits ATP production and 2-DG competitively reduces glucose utilization in the glycolytic pathway. (**B**) ECAR was greater in CTB than in SCT at baseline and was not affected by glucose concentration. (**C**) Maximal glycolytic capacity after oligomycin (O) injection which inhibits ATP production was highest in CTB, but the (**D**) glycolytic reserve was not different between cell types. These data suggest the presence of a substantially greater glycolytic flux in CTB than in SCT under these experimental conditions. (**E**) The ratio of OCR to ECAR at both concentrations of glucose indicates that CTB is more glycolytic than SCT under these conditions. Data are Mean ± SEM, n = 6 placentas. ^#^p < 0.05 vs glucose concentrations.

Glucose is metabolized by the placenta to yield lactate even under conditions where oxygen levels are high. Thus, we tested whether SCT could meet its metabolic needs exclusively through glycolysis by measuring glycolysis in CTB and SCT with extracellular acidification rate (ECAR) as the readout in the Seahorse XF Analyzer. We found significant differences between the ECAR of CTB and SCT (Fig. 3A). Because ECAR is a proxy for lactate production through glycolysis, these experiments suggest that the CTB generates considerable amounts of lactate (Fig. 3A).

ECAR differences were independent of glucose concentration (Fig. 3A). Baseline ECAR in CTB and SCT were (53 ± 5.8 and 18 ± 3.4 ∆mpH × min^−1^ × 100 ng DNA^−1^, p < 0.0001) at 5 mM glucose. The effect of glucose concentration on ECAR could only be detected in CTB at 25 mM glucose (63 ± 5.5 ∆mpH × min^−1^ × 100 ng DNA^−1^ p = 0.04) (Fig. 3B). Maximal ECAR, a measurement of glycolytic capacity, was proportionally greater in CTB compared to SCT (245 ± 10 vs 129 ± 19 ∆mpH × min^−1^ × 100 ng DNA^−1^, p = 0.001). Glycolytic reserve, the difference between baseline glycolysis and maximal glycolysis, was not different between CTB and SCT (Fig. 3D). The relationship between oxidative phosphorylation and glycolysis, the OCR to ECAR ratio, was higher in SCT than in CTB (3.5 ± 0.20 vs.1.3 ± 0.075; p < 0.01) (Fig. 3E), suggesting that SCT “prefers” oxidative phosphorylation under the conditions tested. Although CTB sustain considerable aerobic glycolysis under glucose replete conditions, the CTB OCR is still greater than the SCT OCR under these conditions.

**Figure 4:**
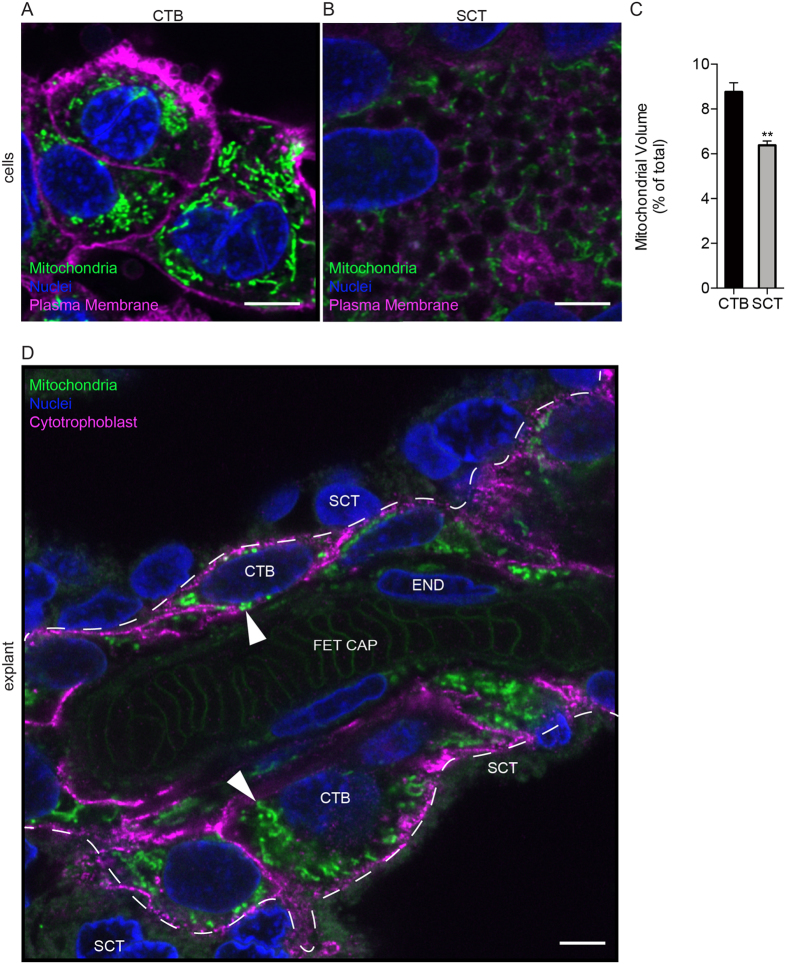
Differentiation of Cytotrophoblast to Syncytiotrophoblast leads to a fragmented mitochondrial network. The mitochondrial activity indicator dye, Mitotracker (CM-H_2_TMRos) was used to localize active mitochondria in CTB and SCT *in vitro* and in living explants of human term placentas using super-resolution fluorescence microscopy. (**A**,**B**) Isolated CTB at 8 hr incubation. Wheat germ agglutinin (magenta) was used to label plasma membrane; nuclei are labeled with Hoechst dye (blue). (**A**) Mitochondria (green) in CTB are tightly packed in a perinuclear fashion. (**B**) The differentiation of CTB to SCT leads to a change in mitochondrial activity, morphology, and to a new arrangement adjacent to cytoplasmic vesicles. (**C**) Mitochondrial volume is relatively larger in CTB than in SCT *in vitro*. (**D**) Human placental explants. Dashed line represents the SCT-CTB interface. In fresh explants of human term placenta, the most active and most brightly stained mitochondria (arrowhead) are located in the CTB layer. A few active mitochondria are also visible in endothelium. CTB in the explants were specifically labeled using HAI-1 (magenta). SCT, Syncytiotrophoblast; CTB, cytotrophoblast; END, endothelium; FET CAP, Fetal Capillary. Scale Bar: 5 μm. Data are Mean ± SEM. Representative images from n = 3 placentas.

Previous investigators have described differences in mitochondrial morphology between CTB and SCT via TEM of tissue sections and isolated mitochondria^15^. We postulated that the differences in mitochondrial activity could underlie our observations of the differences in metabolic activity between CTB and SCT. We used Mitotracker, CM-H_2_TMRosa whose uptake and fluorescence are dependent upon mitochondrial membrane potential (∆ψ) and oxidative activity. In isolated CTB, we found that CTB contain numerous elongated mitochondria in a tightly packed network (Fig. 4A). In contrast, the fewer mitochondria found in SCT were fragmented and distributed around apparent cytoplasmic vesicles (Fig. 4B). The mitochondrial volume ratio in CTB was greater than in SCT (8.7 ± 0.40 vs 6.4 ± 0.19% vol, p = 0.025). We used the Mitotracker method to localize active mitochondria in human term placental explants and found them to be consistent with our studies of isolated trophoblasts. The most active mitochondria were in the CTB layer with low levels in endothelium (Fig. 4C). These data support the view that during the differentiation process as human placental CTB become SCT there are changes in mitochondrial function and morphology and network topology.

**Figure 5:**
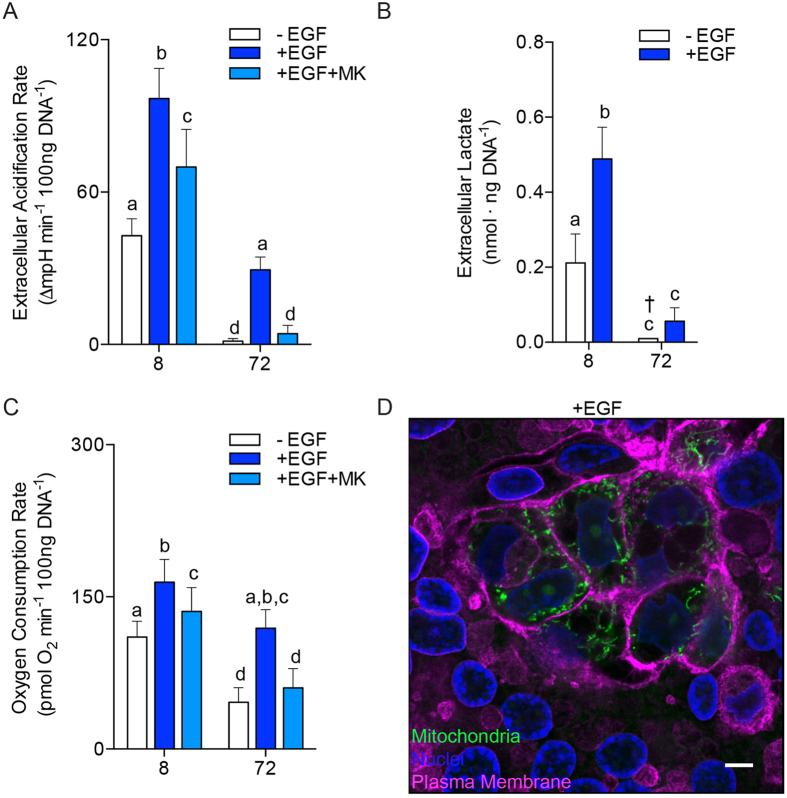
Epidermal Growth Factor and Akt contribute to metabolism and differentiation of cytotrophoblast. Primary human trophoblasts were studied at 8 and 72 h of culture, representing undifferentiated CTB and differentiated SCT in control cultures, respectively. For metabolic measurements, the culture medium was replaced with a Seahorse assay medium supplemented with C16 fatty acids (25 μM) and 5 mM glucose. (**A**) ECAR at 8 h and 72 h of culture, with and without epidermal growth factor (EGF) (10 ng/ml), and with the Akt activation inhibitor, MK2206. (**B**) The higher CTB production of extracellular lactate production over an 8-hour period indicates that CTB are heavily glycolytic and export a large quantity of lactate compared to SCT. Glycolysis and accompanying lactate export in CTB were powerfully stimulated by EGF. MK2206 suppressed the actions of EGF on ECAR. (**C**) OCR was stimulated by EGF, but suppressed by MK2206. (**D**) In the presence of EGF, 72 hr trophoblast cultures contained sheets of SCT, with many aggregates of unfused cells that appeared undifferentiated as indicated by the prominent intercellular membranes (wheat germ agglutinin, magenta). Active mitochondria marked with Mitotracker, CM-H2TMRosa (green) were brightest in the undifferentiated CTB clusters. Scale Bar: 5 μm. Data are Mean ± SEM, n = 6 placentas.

To explore the mechanism leading to metabolic changes in CTB differentiation to SCT, we tested the effect of Epidermal Growth Factor (EGF), a known modulator of the differentiation process^16^. Trophoblast cultures were exposed to EGF in C16 fatty acid supplemented complete growth medium for 8 and 72 h as described previously. We measured baseline glycolysis (ECAR) and respiration (OCR) at both points in time. EGF-exposed trophoblasts at 8 and 72 h had increased rates of glycolysis compared to non-EGF treated trophoblasts [(8 h: 97 ± 12 vs 43 ± 6.7 ∆mpH × min^−1^ × 100 ng DNA^−1^, p < 0.0001); (72 h: 29 ± 5 vs 1.4 ± 1.0 ∆mpH × min^−1^ × 100 ng DNA^−1^, p < 0.01), Fig. 5A].

The data in Figs 3 and 5A indicate CTB cells were producing substantially more extracellular lactate than SCT. However, ECAR is a measure of the rate of pH change and is likely an indirect measure of extracellular lactate production. To test if CTB were producing more extracellular lactate than SCT we directly measured extracellular lactate at 8 and 72 h using an enzymatic assay. These tests supported the data from our previous metabolic flux experiments and clearly demonstrate that lactate production in CTB was much greater than in SCT (8 h: 0.21 ± 0.08 vs 72 h: not detectable nmol × ng DNA^−1^, p = 0.01), Fig. 5B. The latter was undetectable from background. EGF treatment stimulated extracellular lactate production [(8 h: 0.49 ± 0.09 vs 72 h: 0.06 ± 0.04 nmol × ng DNA^−1^, p < 0.001), Fig. 5B]. These experiments indicate CTB glycolytic rate and lactate export is unparalleled and is stimulated by EGF.

Cellular respiration increased both before and after syncytialization with constant exposure to EGF compared to the non-EGF treated state [(8 h: 164 ± 22 vs 96 ± 16 pmol O_2_ × min^−1^ × 100 ng DNA^−1^, p < 0.001); (72 h: 119 ± 18 vs 46 ± 14 pmol O_2_ × min^−1^ × 100 ng DNA^−1^, p < 0.001), Fig. 5C]. In summary, EGF potently stimulated both baseline glycolysis and mitochondrial respiration of trophoblasts by 8 h and this effect persisted to 72 h.

While EGF modulates metabolism via several signaling pathways^17^, Akt, is a central metabolic hub that has been shown to regulate glycolytic rates in many other systems^18^. To test the role of Akt in trophoblast metabolism, MK2206, an Akt phosphorylation inhibitor, was applied. The addition of MK2206 to the EGF-stimulated milieu reduced ECAR by 28% 8 h and 86% at 72 h compared to EGF exposure alone [(8 h: 70 ± 15 vs 97 ± 12 ∆mpH × min^−1^ × 100 ng DNA^−1^, p < 0.01); (72 h: 4.3 ± 3.3 vs 29 ± 5 ∆mpH × min^−1^ × 100 ng DNA^−1^, p = 0.01), Fig. 5A]. MK2206 also led to a smaller decrease in the effect of EGF on OCR at 8 and 72 h [(8 h: 136 ± 24 vs 164 ± 22 pmol O_2_ × min^−1^ × 100 ng DNA^−1^, p = 0.02); (72 h: 60 ± 19 vs 119 ± 18 pmol O_2_ × min^−1^ × 100 ng DNA^−1^, p < 0.001) Fig. 5C]. We did not detect a metabolic difference in trophoblasts with MK2206 in the absence of EGF, suggesting Akt must be activated in order for an MK2206 effect to be detectible.

We reasoned that the metabolic stimulation by EGF at 72 h could have either been due to direct effects of EGF on SCT or EGF could sustain the preservation of CTB in an undifferentiated state. To distinguish these mechanisms, we studied mitochondrial activity using Mitotracker in EGF treated cultures. In the absence of EGF, trophoblast fusion and differentiation formed a syncytial sheet that was virtually complete, and mitochondrial networks became fragmented as described above in Fig. 4. However, unlike non-EGF treated cultures, those treated with EGF contained both fused and unfused cells at 72 h of culture. Interspersed throughout this layer were clusters of individual, unfused, cells with highly active mitochondria (Fig. 5D). These cell clusters contained visible intercellular divisions as found with undifferentiated CTB. Thus, it appears that EGF stimulation maintains a portion of CTB in the unfused condition while promoting fusion among others.

**Figure 6:**
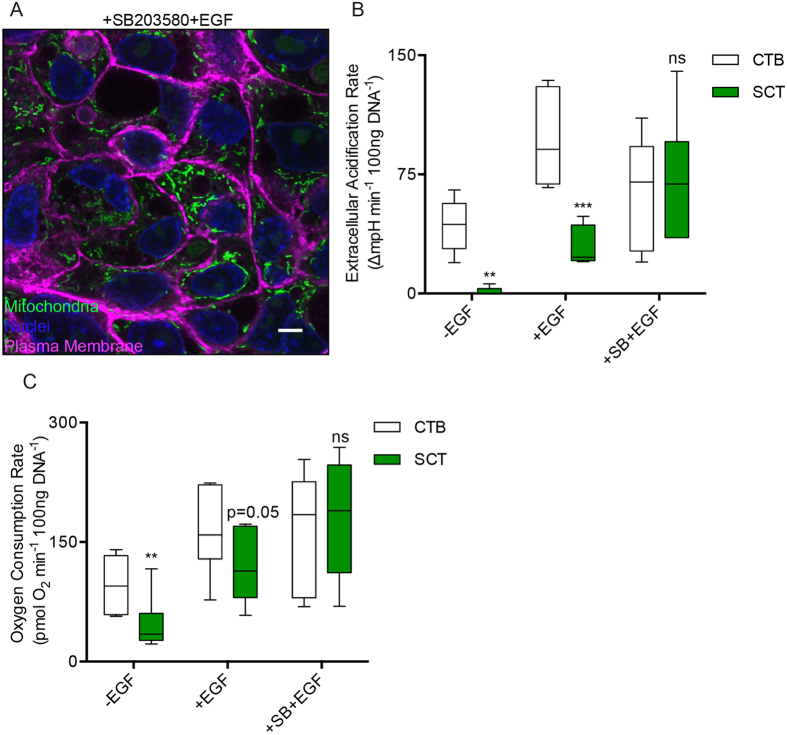
Blocking differentiation of cytotrophoblast preserves their high metabolic rate. (**A**–**C**) The natural course of metabolic suppression during differentiation can be circumvented by employing an inhibitor of the p38 MAPK pathway (10 μM SB203580, SB) coupled with EGF stimulation. (**A**) Confocal micrograph of CTB treated with EGF and SB203580 for 72 h, the time point when normal cytotrophoblast cells have fused, and stained with Mitotracker (green) and (wheat germ agglutinin, magenta). Cell fusion does not occur under these conditions, and all cells have active mitochondria. CTB fusion was blocked with this combined treatment and active mitochondria can be observed in all cells. (**B**) ECAR and (**C**) OCR, are elevated and maintained between 8 and 72 h when treated with the combination EGF and SB203580. Scale Bar: 5 μm. Data are Mean ± SEM, n = 6 placentas.

To uncouple the metabolic effects of EGF with its effect on trophoblastic differentiation, we blocked the differentiation of CTB while exposed to EGF with the p38 MAPK inhibitor, SB203580^16^. As expected, blocking the differentiation program in CTB by combining SB203580 with EGF inhibited CTB fusion over 72 h of culture (Fig. 6A) and maintained high levels of ECAR and OCR, which was characteristic of undifferentiated CTB. Thus, there was no apparent decrease in ECAR or OCR between 8 and 72 h [(8 h ECAR: 64 ± 14 vs 72 h ECAR: 71 ± 16 ∆mpH × min^−1^ × 100 ng DNA^−1^, p = n.s.); (8 h OCR: 165 ± 31 vs 180 ± 30 pmol O_2_ × min^−1^ × 100 ng DNA^−1^, p = n.s.), (Fig. 6B,C)]. These results contrast with control cultures and those exposed to EGF only, where marked metabolic suppression occurs during trophoblast fusion by 72 h of culture (Fig. 6B,C).

CTB treated with SB203580 medium alone did not maintain the high levels of glycolysis or oxidative respiration that usually characterizes CTB *in vitro* (72 h ECAR: 1.9 ± 1.2 vs 1.4 ± 1.0 ∆mpH × min^−1^ × 100 ng DNA^−1^, SB203580 vs control; p = 0.99); (72 h OCR: 45 ± 6.9 vs 32 ± 3.8 pmol O_2_ × min^−1^ × 100 ng DNA^−1^ SB203580 vs control; p = 0.86), Fig. 6B,C. These results suggest that EGF is necessary for maintenance of the CTB metabolic phenotype under the conditions of these experiments.

## Discussion

We have provided several lines of evidence that CTB is the most metabolically active cell in the human placenta at term, which contradicts the prevailing view that SCT is the dominant metabolic cell^9^,^14^,^19^. We measured rates of mitochondrial respiration and glycolysis in primary human trophoblast as undifferentiated progenitor cells (CTB) and after differentiation to SCT. We consistently found greater levels of both glycolysis and mitochondrial respiration in CTB compared to SCT. In addition, ATP levels were higher in CTB. Higher levels of ATP in CTB could be due to a greater synthetic rate and/or reduced utilization. The most fascinating finding was that CTB is the more metabolically flexible of the two cell types. CTB could better maintain respiratory rates under varying nutrient levels in the growth medium during our stress tests. Nevertheless under all conditions tested, we consistently found the ratio of OCR to ECAR to be less in CTB than SCT (Fig. 3E). This suggests that compared to SCT, CTB are more glycolytic relative to their respiration rate.

The placenta utilizes aerobic glycolysis as its primary metabolic fuel to conserve oxygen supplies for fetal tissues and it produces lactate which is a critical fuel source for fetal growth^2^,^20^. The placenta is known to be the primary production site for lactate in the fetus^21^,^22^. Approximately 25% of the CO_2_ produced by the fetus comes from the oxidation of lactate that was delivered to it from the placenta^21^. Our data suggest that placental glucose consumption and lactate production are principally driven by the CTB rather than SCT. A key exporter of lactate, the monocarboxylate transporter 1 (MCT1), is highly expressed in the CTB of human placentas^23^. It is not known if the CTB participates in transplacental nutrient transport, but the high metabolic activity and strategic placement of the CTB layer adjacent to the basal SCT plasma membrane suggest that the CTB layer could be important not only for generating ATP and lactate but also in nutrient transport and processing^12^,^24^.

Lactate production by CTB could be associated with anabolic metabolism. Aerobic glycolysis and lactate production support *de novo* lipogenesis^25^, including synthesis of cholesterol, which is key for metabolic activity in proliferating cells. CTB, but not SCT, are proliferative and CTB cells have much greater rates of *de novo* lipogenesis than SCT^26^.

Previous reports also support the idea that CTB is a highly metabolically active placental cell^14^,^27^ and this suggestion is not exclusive to the term placenta^28^. However, to our knowledge this study is the first to directly compare the oxidative and glycolytic flux rates of CTB from term human placentas as they differentiate and become SCT. Other studies on isolated mitochondria from the placenta support our findings that CTB mitochondrial respiration is greater than SCT^27^.

Our findings are relevant to the numerous studies that have found alterations in placental metabolism in preeclampsia^5^,^6^ and obesity^7^. Indeed, abnormal CTB differentiation and EGF signaling have been proposed as important in the pathophysiology of preeclampsia^29^,^30^. Glycolysis rates and lactate production are lower in placentas from preeclamptic pregnancies compared to normal placentas^6^ and impaired placental fatty acid oxidation has been proposed to underlie the pathophysiology of preeclampsia^31^,^32^, which we have shown herein to be higher in CTB than SCT. In the studies reported here, we have shown that CTB metabolism can be regulated via EGF and p38 MAPK signaling.

EGF signaling stimulated the metabolic rate of CTB and rescued the suppression in metabolism during the normal course of CTB differentiation to SCT. Johnstone *et al*. demonstrated EGF treatment of term CTB inhibits HCG production, increases CTB proliferation approximately 2-fold, and increases the number of multinucleated giant cells. Since the formation of multinucleated trophoblast is a function of cell density, an increase in trophoblast density due to proliferation could result in the increase in multinucleation^16^. We are intrigued that groups of undifferentiated cytotrophoblast cells remain adjacent to fully syncytialized trophoblast in culture following EGF treatment, as occurs in living human placentas. We speculate that EGF is required for normal metabolism of trophoblast. Mouse, primate, and human studies have suggested that fetoplacental growth can be regulated throughout pregnancy by the EGF axis^33^,^34^,^35^.

Some of the direct metabolic effects of EGF could be attributed to Akt kinase signaling in CTB, and Akt activity may contribute to the proliferation and/or differentiation of CTB^36^. We tested the hypothesis that blocking differentiation of CTB with a p38 MAPK inhibitor (SB203580) could circumvent the metabolic suppression observed in the transformation of CTB to SCT. It did not. However, the combination SB203580 and EGF treatment greatly stimulated and maintained the high levels of metabolism over the time frame that CTB would ordinarily differentiate. By 72 h, trophoblast treated with this combination exhibited metabolic fluxes similar to undifferentiated CTB measured at 8 h, and these metabolic rates were greater than 72 h cultures treated with EGF alone. These observations are consistent with other studies demonstrating that the p38 MAPK pathway modulates the ability of trophoblast to respond to growth factors, including EGF^16^ and serum^37^.

It appears that fragmentation of the mitochondrial network is associated with changes in metabolism as CTB fuse to become SCT. CTB mitochondria are larger, less dense, and contain abundant cristae^15^, and SCT mitochondria remodel, perhaps in preparation for steroidogenesis by becoming fragmented, smaller, more dense and modified to lose identifiable cristae^15^,^38^,^39^. While even our super-resolution methods cannot resolve mitochondrial cristae, we found that the mitochondrial network decreased in density and appeared fragmented after differentiation.

We measured the respiration and glycolysis of the two main trophoblast cell types in human placenta, CTB and SCT, but have not yet compared these to the other cells in placenta, e.g. stroma and endothelium. The experiments using the fluorescent mitochondrial activity reporter indicate that these other placental cell types have much lower mitochondrial membrane potentials and/or lower mitochondrial oxidative activity. Future studies should aim to directly quantify the metabolic rates of placental stroma and endothelium in addition to CTB and SCT.

In conclusion, we present multiple lines of evidence that the metabolic activity of the placenta is driven not only by the SCT but by the underlying CTB as well. Until now, the relevance of the CTB to human placental metabolism has not been appreciated. We suggest that future studies of placental biology should include comparisons of both CTB and SCT. These two cell types are highly specialized to serve unique and crucial roles during development that are only now being discovered. Our study focused on understanding normal metabolic rates of CTB compared to SCT, but future studies will test if pathological conditions like preeclampsia, diabetes mellitus, or fetal growth restriction lead to changes in CTB metabolism and additionally EGF signaling. The regulators of placental metabolism is of great importance as we are currently clinically limited in our understanding of the true pathophysiology of abnormal metabolic conditions as in the placenta. Because fetal growth restriction carries a significant risk for neonatal and adult onset disease^40^,^41^, gaining more insight into how placental cell types regulate nutrients and fuels required for fetal growth and development should be a high priority.

## Materials and Methods

### Subject Details

This study was approved by the Institutional Review Board (IRB #5684), the methods were carried out in accordance with all relevant guidelines and regulations, and informed consent was obtained prior to delivery. Placentas were collected from women undergoing a scheduled cesarean delivery at the Oregon Health & Science University (OHSU) obstetric unit from 06/2015 to 05/2016. Uncomplicated, singleton placental samples were included, excluding pregnancies complicated by the following characteristics: prepregnancy obesity (BMI ≥ 30 kg/m^2^), multifetal gestation, fetal chromosomal or structural anomalies, preeclampsia or hypertensive spectrum, diabetes mellitus, immunosuppressive, or chronic maternal morbidities. Maternal data were obtained from medical records included age, parity, race, gestational age at delivery, height, and weight (1st trimester). Neonatal data included birth weight, crown-heel length, and sex. Placenta weight and length and width dimensions were measured during tissue collection. Maternal characteristics are summarized in Table 1.

### Primary human villous cytotrophoblast isolation and culture

All placental samples were processed within 30 minutes of delivery. CTB cells were isolated using a trypsin-DNAse I digestion followed by Percoll enrichment as previously described^42^. In brief, chorion and maternal decidua were removed, and 40–50 g of villous placental tissue was finely minced and thoroughly washed with 1x phosphate-buffered saline (PBS). Villous fragments were subjected to three or four sequential 10–15 minute (37 °C) digestions in 0.25% Trypsin (Gibco) and 200 U/mL DNAse I (Roche). CTB were purified by Percoll (GE Healthcare Bio-sciences AB) discontinuous density gradient centrifugation at 1200 rcf for 25 minutes (room temperature). Purity of trophoblast isolations was assessed by positive immunohistochemical staining of cyokeratin-7 (MAI-06315, Thermo Scientific), a marker of trophoblast cells. All isolations comprised of >90% pure, viable CTB.

CTB were plated at an optimal density of 3 × 10^5^ cells/cm^2^ and cultured in Iscoves Modified Dulbecco’s Medium (IMDM, Gibco^®^) or Minimum Essential Media alpha GlutaMAX™ without nucleosides (MEMα, Gibco^®^) supplemented with 10% fetal bovine serum (FBS, Gibco^®^), 100 U/mL penicillin, and 100 μg/mL streptomycin and incubated at 37 °C 5% CO_2_. Growth medium was replaced every 24 hours. Prior studies have demonstrated CTB undergo fusion and differentiation into multinucleated, syncytialized giant cells by 72 h in culture^10^. Therefore, cells were studied at 8 hours (CTB) to study trophoblasts prior to differentiation and 72 h to study differentiated, syncytialized cell types (SCT).

### Metabolic Flux Analyses

Primary human trophoblast cells (40,000/well) were plated into 96-well culture plates. The oxygen consumption rate (OCR) and extracellular acidification rate (ECAR) were measured using the Seahorse XF^e^96 flux analyzer (Seahorse Bioscience, North Billerica, MA). At the time of assay, growth medium was removed, replaced with the appropriate pre-warmed Seahorse XF Assay Medium (Seahorse Bioscience), and cultures underwent a Mitochondria or Glycolytic stress test™ (Seahorse Bioscience).

For fatty acid oxidation studies, assay medium was composed of warmed serum-free 1x Krebs Henseleit Buffer (KHB) supplemented with 0.5 mM glucose, 0.5 mM carnitine and 5 mM HEPES (pH 7.4). Fatty acid: Bovine Serum Albumin (BSA) (~6:1) conjugates were prepared according to the manufacturers recommendations and supplemented to KHB assay medium^43^. Immediately prior to assay measurements fatty acid supplements were added with the following final concentrations C16 [250 μM], C18:1 [250 μM] or vehicle (BSA [41 μM]) +/− etomoxir (Eto 40 μM), a carnitine palmitoyltransferase 1a inhibitor (CPT1a) of fatty acid oxidation. Under these conditions the following injections and final concentrations were: (1) 2 μM oligomycin (ATP synthase inhibitor; Sigma), (2) 5 μM carbonyl cyanide p-trifluoromethoxyphenyl-hydrazone (FCCP) (mitochondrial uncoupling agent; Sigma), and a (3) mixture of 2 μM Rotenone (mitochondrial complex I inhibitor; Sigma) and 2 μM Antimycin A (Mitochondrial complex III inhibitor; Sigma). OCR was recorded for three cycles following each timed injection.

For glycolytic stress test measurements, XF Base Medium (Seahorse Biosciences) was supplemented with 1 mM pyruvate and 4 mM L-glutamine. The Glycolytic stress test injections and final concentrations included: (1) 5 or 25 mM Glucose, (2) 1 μM oligomycin, (3) 100 mM 2-Deoxyglucose (hexokinase inhibitor; Sigma). ECAR was recorded for three cycles following each timed injection.

The metabolic influences of Epidermal growth factor (EGF) were studied. CTB were cultured in MEMα medium in the presence or absence of the following: 10 ng/mL EGF, 1 μM of MK 2206 (allosteric inhibitor of protein kinase Akt), 10 μM SB203580 (p38β MAPK inhibitor). After 8 and 72 hours of culture, growth medium was removed and replaced with an assay medium containing glucose (5 mM), glutamine (4 mM), pyruvate (1 mM) and C16 fatty-acid (25 μM), measuring full metabolic capacity. Four serial baseline OCR and ECAR measurements were analyzed and averaged. The metabolic flux capacity was measured in triplicate for each condition, replicated for at least an n = 6, and all experimental measurements were normalized to DNA content using Quant-iT™ PicoGreen^®^ dsDNA kit (Molecular Probes; Eugene, OR).

### Intracellular ATP and Extracellular Lactate quantifications

Isolated CTB were plated on flat-bottom 96-well plates as previously described and cultured in complete growth medium for 8 and 72 hours. The growth media was removed and 40 μL of the cell lysate was used to quantify intracellular ATP using an ATP luminescence assay (ATPlite^™^, PerkinElmer^®^). Luminescence was measured using a Biotek Synergy H1 plate reader (Biotek). ATP content was normalized to lysate protein concentration using a bicinchoninic acid assay assay (Thermo Pierce).

To measure extracellular lactate, medium was collected from CTB incubated in 96-well plates as described with fresh medium during an 8-hour period, representing lactate produced during 0–8 h or 64–72 h in culture. Extracellular lactate was measured using a colorometric lactate assay kit (Sigma) and plates were read using a Biotek Synergy H1 plate reader. Lactate measurements were normalized to DNA content using the Quant-iT™ PicoGreen^®^ dsDNA kit as outlined for metabolic flux analyses.

### Mitochondrial labeling, imaging, and quantification

Isolated CTB were plated on glass coverslips and cultured for 8 or 72 hours in complete growth medium. At each specified time point, 500 nM of Mitotracker Orange (CM-H_2_TMRosa; Molecular Probes) was added to each well and incubated for 2 hours. Twenty minutes prior to the completed incubation period, 4 μg/ml Wheat Germ Agluttinin-CF640R (Biotium) was added to demarcate the plasma membrane. After 2 hours, cells were fixed and incubated with pre-warmed 3.7% paraformaldehyde (pH 7.4) in complete growth medium for 30 minutes at 37 °C/5% CO_2_. After fixation, the coverslips were submersed into −20 °C Acetone for 10 minutes and subsequently washed up to 3 times in 1x PBS. The coverslips were counterstained with 2 μg/ml Hoechst 33258 (Molecular Probes) to label nuclei for 20 minutes and mounted in Slowfade Diamond (Molecular Probes, Inc) before confocal imaging.

Imaging was performed using a Zeiss 880 LSM Confocal with Airyscan™ with a 63× High N.A. objective (N.A. = 1.4; Zeiss). Each field of view was comprised of serial Z-“stack” of images measuring 67.5 μm by 67.5 μm (x-y), spaced 0.2 μm apart and 2 μM total thickness. All raw, 32-channel single-color images were processed using automatically determined Airyscan parameters in Zen software (Zeiss). Processed images were analyzed using Fiji software^44^ and Otsu automatic thresholding to segment mitochondria. The mitochondrial volume ratio was calculated by normalizing the quantified mitochondrial volumes to cytoplasmic volumes quantified from plasma membrane images. A minimum of 3 fields of view were used per biological replicate.

### Placental explant collection, culture, and imaging

Explants (<1 mm^3^) were isolated as previously described^45^, with some modifications. Mitochondrial labeling proceeded immediately after placental collection (<30 min) as described above for cell isolation. Transwell permeable supports (Corning) were used in 12-well plastic culture plates (Corning) for culture. Each well contained three to four tissue explants from different, healthy appearing cotyledons and were cultured in 2.0 mL of pre-warmed (37 °C) MEMα culture media (Gibco) supplemented with fetal bovine serum and 25 mM HEPES (pH 7.4), incubated at 37 °C in 5% CO_2_/95% air for 2.5 hours. Explants were assayed within a 2 hour window when markers of explant health and nutrient uptake are not compromised^45^.

### Immunofluorescence (whole mount explants, cells)

Fixed explants and cells were blocked and permeabilized using Block-Aid (Life Technologies)/0.1% Tween-20 (ThermoFisher) for 30 minutes before overnight incubation with primary antibody at 4 °C. Samples were labeled with antibodies against HAI-1 (1:100; 9B10, eBioscience). Following primary antibody incubation, samples were washed 3 times with 0.01% Tween-20 in PBS and labeled with secondary antibodies rabbit anti-mouse Alexa Fluor 488 (1:500, A27023, Invitrogen) for 1 hour at room temperature. After washing 3 times, the samples were counterstained with 2 μg/ml Hoechst 33258 for 20 minutes and immersed in Slowfade Diamond (Molecular Probes) immediately before confocal imaging.

### Quantification of mRNA expression

Total RNA from 3 × 10^6^ cells was isolated after 8 hr or 72 hr of culture using Qiagen RNeasy isolation kit. RNA content was assessed by spectroscopy at 260 nm/280 nm and integrity via visualization of ribosomal RNA using gel-electrophoresis. Reverse transcription of 1 μg of RNA to cDNA was performed using the High Capacity cDNA Reverse Transcription kit (Life Technologies). cDNA was stored at −20 °C. Gene specific primers were designed for VPS29 house-keeping gene (primer sequences: F GACAGGATGTTGGTGTTGGT, R TAGCTGGCAAACTGTTGCAC) using NCBI primer-BLAST^46^. Sequences for primers for BHCG (*CGB*) taken from Kolahi *et al*.^12^ qPCR was performed as previously described^47^. Relative expression quantities were expressed as a ratio of the gene of interest to the reference gene (*VPS29*) in each sample; *VPS29* expression did not differ at 8 hours versus 72 hours.

### Statistics

2-way ANOVA with Sidak’s post-hoc testing was used to compare metabolic fluxes between conditions. An unpaired Student’s t-test was used to compare mitochondrial volume ratios. All data were analyzed using GraphPad Prism 6 and are presented as mean ± SEM unless noted otherwise. P-values < 0.05 were considered statistically significant.

## Additional Information

**How to cite this article:** Kolahi, K. S. *et al*. Cytotrophoblast, Not Syncytiotrophoblast, Dominates Glycolysis and Oxidative Phosphorylation in Human Term Placenta. *Sci. Rep.* **7**, 42941; doi: 10.1038/srep42941 (2017).

**Publisher's note:** Springer Nature remains neutral with regard to jurisdictional claims in published maps and institutional affiliations.

## Figures and Tables

**Table 1:** Maternal Characteristics.

	Mean ± S.D. (n = 28)	95% Confidence Interval
Age (yr)	31 ± 5	[28, 34]
Body Mass Index (BMI: kg/m^2^)	23 ± 5	[20, 26]
Parity	2 ± 2	[1, 3]
Gestational Age (weeks)	39 ± 1	[39, 40]
Birth Weight (g)	3300 ± 550	[3000, 3700]
Placenta Weight (g)	493 ± 104	[429, 557]
